# A national survey into perioperative anesthetic management of patients with a fractured neck of femur

**DOI:** 10.1186/1471-2253-12-14

**Published:** 2012-07-28

**Authors:** Mirka Soinikoski, Kristiina Kuusniemi, Jouko Jalonen, Sari Kuitunen, Tapani Tuppurainen, Kari Leino

**Affiliations:** 1 Department of Anesthesia, Intensive Care Medicine, Emergency Medicine and Pain Medicine, Turku University Hospital, Turku, Finland; 2 Department of Anesthesia, Intensive Care Medicine, Emergency Medicine and Pain Medicine, University of Turku and Turku University Hospital, Turku, Finland; 3 Medical E-learning, Faculty of Medicine, University of Turku, Turku, Finland; 4 Department of Anesthesia, Intensive Care Medicine, Emergency Medicine and Pain Medicine, Turku University Hospital, Kiinamyllynkatu 4-8, 20520, Turku, FI-20520, Finland

**Keywords:** Hip fracture, Perioperative management, Anesthetic practice

## Abstract

**Background:**

We made a survey among Finnish anesthesiologists concerning the current perioperative anesthetic practice of hip fracture patients for further development in patient care.

**Methods:**

All members of the Finnish Society of Anesthesiologists with a known e-mail address (786) were invited to participate in an internet-based survey.

**Results:**

The overall response rate was 55% (423 responses); 298 respondents participated in the care of hip fracture patients. Preoperative analgesia was mostly managed with oxycodone and paracetamol; every fifth respondent applied an epidural infusion. Most respondents (98%) employed a spinal block with or without an epidural catheter for intraoperative anesthesia. Midazolam, propofol and/or fentanyl were used for additional sedation. General anesthesia was used rarely. Postoperatively, paracetamol and non-steroidal anti-inflammatory drugs and occasionally peroral oxycodone, were prescribed in addition to epidural analgesia.

**Conclusions:**

The survey suggests that the impact of more individualised analgesia regimens, both preoperatively and postoperatively, should be investigated in further studies.

## Background

Hip fracture is a common injury and the leading fall-related cause of death among the elderly patients, with significant 30-day and one-year mortality rates [1-3]. These patients constitute a significant workload, not only to operating departments and the surgical ward, but to the whole health care system. Given the rapidly growing amount of elderly people in the Western world, the management of these patients will become increasingly important in the future. The perioperative care of these patients is also becoming more complex with a growing amount of patients with a number of specific medications for concurrent diseases and for the prevention of thromboembolism. The anesthesiologist must take these into account when planning anesthesia and analgesia techniques.

Sandby-Thomas et al. [4] conducted a national survey into the perioperative anesthetic management of hip fracture patients in the UK in 2006. Due to a somewhat different practice of using regional anesthesia and analgesia techniques compared with the UK we conducted a corresponding survey among Finnish anesthesiologists. Our aim was to characterize the current status of the anesthetic and analgesic practice in Finland for further development in patient management strategies. The major interest was in the answers of individual anesthetists.

## Methods

We conducted this national survey in February 2009 via internet by using an electronic questionnaire (Webropol, Helsinki, Finland, http://w3.webropol.com). Our research did not involve human subjects including human material or human data. We were informed by the Finnish Medical Association (FMA) and its lawyer that an approval of an appropriate ethics committee or any consent would not be implied on this occasion. A basic activity of the Finnish Medical Association throughout its existence has been to promote ethical principles. Finland was the first country in Europe to enact legislation relating to the status and rights of patients. In response to an initiative taken by the FMA, the World Medical Association (WMA) has adopted the Declaration on the Rights of the Patient which is binding on the medical profession in every country. The FMA also made significant contributions to the amendment of the Ethical Principles for Medical Research Involving Human Subjects (the Declaration of Helsinki). Based on these declarations, we were advised by the FMA lawyer that we wouldn't need any further approvals from the ethics committee or consequently any consent since we were doing a national survey concerning the opinions and treatment practices of the members of the Finnish Society of Anesthesiologists.

We obtained a list of all the members of the Finnish Society of Anesthesiologists from the General Secretary of the Society. The society had 989 active members at the time of the survey. Of the active members, 772 were specialists and 217 were trainees in anesthesia and intensive care. We sent an invitation to participate in the survey by an e-mail message to all the members with a known e-mail address, altogether 768 anesthetists. The invitation was resent twice (with a three-week interval) to assure that all the members had received the message and had an opportunity to answer. The Webropol platform allows only one reply from each e-mail address, thus preventing multiple replies from a single person.

The survey was in a structured tick box format and it also provided free text fields for each group of inquired items for more detailed answers and explanations. The questionnaire was divided into three main sections: 1) preoperative pain management, 2) anesthetic technique and intraoperative analgesia and sedation, and 3) postoperative pain management. The type of respondent’s hospital (university, central, regional or other) and respondent’s specialist/trainee status were also inquired. The frequency of their use of a particular technique was graded on a five-step scale: always, mostly, occasionally, rarely and never. Results of the survey are expressed as the percentage and the number of anesthetists using a particular technique always or mostly per all individuals who had responded to that individual question.

## Results

### Respondents

The invitation was sent to 768 anesthesiologists, of whom 423 replied, producing an overall response rate of 55%. However, 125 respondents answered that they did not treat hip fracture patients, leaving 298 who did. The response rate was 79% among specialists and 21% among trainees. All who responded did not, however, answer all questions; the median frequency by which each question was answered was 292 (range 151–295). Most respondents worked either in a university hospital (53%) or in a central hospital (28%).

### Preoperative care

**Figure 1:**
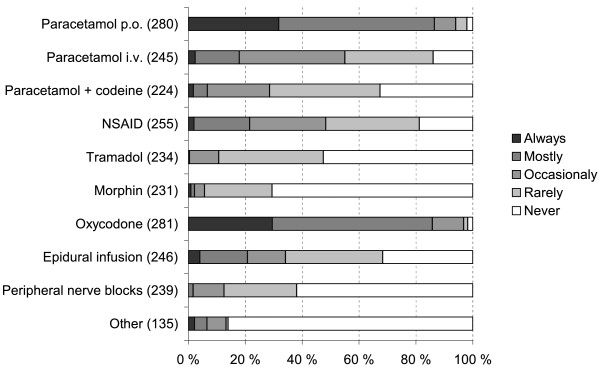
Use of preoperative analgesic techniques in hip fracture patients among respondents (altogether 292 respondents; number of respondents for each item in parentheses).

Oxycodone and paracetamol (acetaminophen) were preferred in the use of preoperative pain management, while epidural infusion and non-steroidal anti-inflammatory drugs (NSAIDs) were employed less often (Figure 1).

### Intraoperative care

**Figure 2:**
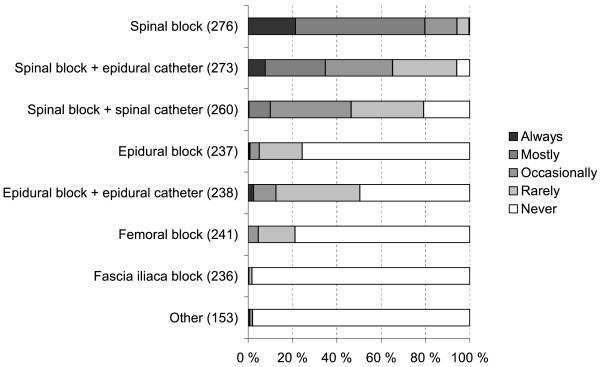
In intraoperative care, the use of various regional anesthesia techniques in hip fracture patients when using only regional anesthesia (altogether 293 respondents; number of respondents for each item in parentheses).

**Figure 3:**
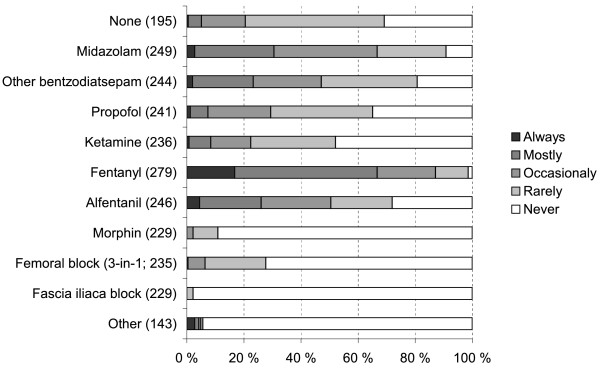
In intraoperative care, the use of additional preoperative medication with regional anesthesia in hip fracture patients (altogether 292 respondents; number of respondents for each item in parentheses).

Regional anesthesia alone was used always or mostly among 98% (286/291) of the respondents. A vast majority of the anesthesiologists who responded employed a spinal block or a spinal block in conjunction with an epidural catheter (Figure 2). A majority of respondents gave some adjunctive medication, mostly intravenous fentanyl or alfentanil, to facilitate positioning for spinal and epidural anesthesia (Figure 3). The patient was placed almost exclusively with the injured side upwards (always or mostly by 294/294 respondents). The amount of bupivacaine used in the spinal injection was commonly 2.5 -3.5 ml by 62% (181/276 of respondents) but quite many employed doses below 2.5 ml (43%, 113 of 266 respondents); 53% of the respondents (129/243) never used a volume > 3.5 ml. 98% (286/292) of the respondents used plain isobaric bupivacaine and 24% (61/258) added fentanyl to the spinal injection. Intraoperatively, when additional sedation was required, midazolam, propofol or/and fentanyl were administered. Ketamine was used rarely and 26% (53/205) responded that they always or mostly administrated no additional sedative at all.

General anesthesia alone or combined with a spinal block with or without an epidural catheter was used rarely. When using combined general and regional anesthesia, 56% (112 of 200 respondents) inserted an epidural catheter and 40% (77/192) of the respondents used a spinal block. A spinal catheter, a femoral nerve block or local infiltration analgesia (LIA) were used occasionally. In patients receiving general anesthesia, 79% (200 of 252 respondents) used always or mostly tracheal intubation, 83% (207/251) used controlled ventilation and 65% (161/249) used neuromuscular blocking drugs. A laryngeal mask airway (LMA) was used rarely or never. Inhalation anesthesia was chosen by 83% (211/254 of respondents) for maintenance of general anesthesia.

### Postoperative care

**Figure 4:**
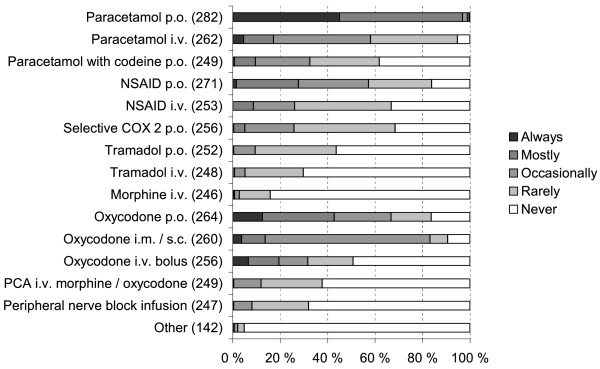
Use of additional postoperative analgesia at the ward in hip fracture patients when the patient had an epidural infusion (altogether 282 respondents; number of respondents for each item in parentheses).

**Figure 5:**
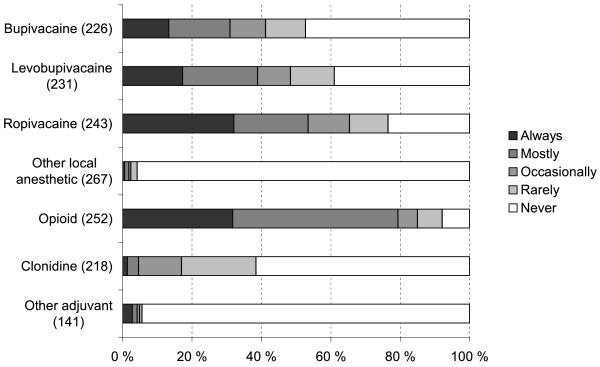
Regimens used in postoperative epidural infusion in hip fracture patients (altogether 270 respondents; number of respondents for each item in parentheses).

**Figure 6:**
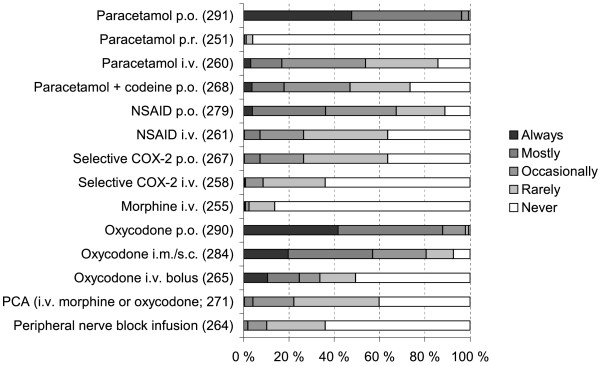
Postoperative analgesia at the ward in hip fracture patients when the patient did not have an epidural infusion (altogether 295 respondents; number of respondents for each item in parentheses).

If the patient had an epidural infusion, most respondents additionally used paracetamol, NSAIDs and oxycodone to treat postoperative pain, all administered primarily orally (Figure 4). Postoperative epidural infusion usually contained ropivacaine, levobupivacaine or bupivacaine combined with an opioid adjuvant (Figure 5). If the epidural infusion contained an opioid adjuvant, 84% (232 of 276 respondents) did not prescribe other opioids. Femoral nerve block was used rarely for postoperative analgesia. If the patient did not have an epidural infusion, 96% (280/291) of the respondents administered always or mostly peroral paracetamol and 88% (255/290) prescribed peroral oxycodone (Figure 6).

## Discussion

We achieved a reasonable response rate enabling us to assume that the present survey represents the current anesthetic practice in Finland accurately enough. This study confirms the clinical impression of spinal block being the most commonly used method for anesthesia for hip fracture surgery, with only a few physicians using general anesthesia. Preoperative and postoperative pain management was characterised by a common use of oxycodone in conjunction with paracetamol, while epidural infusion was applied preoperatively only by every fifth respondent. There is an evident need for more advanced strategies for the perioperative care of hip fracture patients and we hope that this survey will warrant further research to develop the current care.

### Preoperative analgesia

Roughly estimated only every fifth respondent used an epidural infusion preoperatively. Given the available data that endorses the application of preoperative epidural infusions we would have expected a somewhat wider use. Effective analgesia provided by a preoperatively started epidural infusion reduces perioperative myocardial ischemia [5,6]. The risk of death, prolonged hospitalisation and long-term institutionalisation is particularly high in patients with early confusion or dementia [7,8]. Delirium was also frequently reported in the free text fields of the present survey. Prevention and using a multi-factorial intervention program has been shown to reduce the incidence of delirium [9]. Surgery and effective rehabilitation should start without delay to allow ambulation after surgery as early as possible. According to our survey the preoperative analgesia of the hip fracture patients could be further intensified when compared with these evidence-based data [5,6,9]. It is known that a delay in the operation after admission may increase mortality [10] and therefore aggressive care during the eventual waiting period may be critical. On the other hand, required preoperative optimisation of complex co-morbidities may cause delay [11].

### Intraoperative care

A vast majority of anesthesiologists preferred spinal block, epidural block or a combination of these two as the method of anesthesia, instead of peripheral neural blocks and general anesthesia. Regional anesthesia was also the most preferred one by trauma anesthetists in the UK [4], though we had even a greater share of spinal blocks compared with them. Unlike in the UK, in Finland the patients were almost exclusively positioned the affected side upwards. Consequently, there was also a great difference in the use of plain and heavy bupivacaine for spinal anesthesia between the two countries. It is worth noticing that our survey was conducted almost three years later than that of Sandby-Thomas [4] and this may have some contribution to the differences. There is a discrepancy on whether regional anesthesia provides better outcome than general anesthesia in patients with a hip fracture repair [12-14]. However, according to the (debated) review of Rodgers et al. [15] especially orthopaedic surgery and spinal anesthesia were associated with decreased mortality compared with general anesthesia, and even more recent studies and reviews recognise superior analgesia, lesser metabolic stress response, better maintained bowel motility and less respiratory problems with neuraxial techniques compared with general anesthesia [13,15]. Probably even a greater share of patients would have been treated with a central block if there had not been obvious contraindications, especially disturbed hemostasis due to anticoagulant and antithrombotic treatments. In fact, our current clinical practice may be a subject to change as we probably will more frequently encounter an increasing variety of antithrombotic drugs, as the combined use of these drugs and a central neuraxial block predisposes patients to spinal hematoma, a rare but serious complication. This issue is of increasing importance and it was addressed recently by the Scandinavian Society of Anesthesiology and Intensive Care in form of Nordic guidelines [16]. These guidelines reflect the complexity when determining whether a patient can or cannot be safely anesthetised using a central block and how to manage these drugs postoperatively.

### Postoperative analgesia

Postoperative analgesia was commonly accomplished with an epidural infusion of a local anesthetic agent, most commonly bupivacaine, alone or together with an opioid. Other analgesic modalities included administration of paracetamol, NSAIDs and systemic opioids. Simultaneous use of epidural and systemic opioids is not commonly recommended due to the fear of excessive respiratory depression. According to our survey, this practice was not entirely uncommon, but our data did not allow exploring the eventual respiratory effects of this practice.

Compared with other European countries, oxycodone is a more commonly used systemic opioid in Finland than morphine. Oxycodone is a μ agonist, by and large comparable to morphine, with relatively predictable analgesic effect even by oral administration [17,18]. However, it does have the same common undesirable effects as morphine, in elderly patients especially nausea and constipation, which may delay mobilisation and recovery. Apparently for this reason, according to our survey, other systemic analgesics like paracetamol and NSAIDs were commonly used as first-line analgesics and as adjuvants of neuraxial techniques. However, the relatively high prevalence of renal dysfunction among elderly patients may contraindicate the use of NSAIDs in some patients, while opioids may induce respiratory depression and increase mortality [17]. Furthermore, a shift from opioid analgesia to femoral nerve block techniques, as a part of the optimised hip fracture program, has reduced the rate of in-hospital postoperative complications and mortality [19]. Even in an optimised hip fracture treatment program each patient must be treated individually, since postoperative pain intensity varies after different types of repair techniques [20].

This type of survey cannot address all contributing factors like co-morbidities, impaired mental and physical capacity, medication and drug interactions that may well be highly important for the outcome of a hip fracture patient. For example, hip fracture patients with delirium compared with other patients have higher mortality rates, a greater risk of institutionalisation and a worse prognosis for recovery [10,11,19,21]. Trauma, anesthesia, dehydration, fluid balance disturbances, fever, sepsis and changes in cardiac function may also alter the pharmacokinetics of drugs [22]. Controlled intervention studies are clearly needed to tailor recommendations for the holistic treatment of this particularly challenging group of patients [23,24].

### Limitations

We believe that our approach to survey the practices of individual anesthesiologists reflects the frequency how individual patients are subjected to the use of a certain technique. However, the number of cases of interest may vary for an individual anesthesiologist, and no attempt was made to estimate the weight of the answers of individual respondents. Also, a web-based survey does not allow comparing used techniques and the outcome. Further, an e-mail invitation can only reach those who have an updated e-mail address and who regularly use e-mail. This had probably some effect on the expected response rate (which was calculated for all registered Society members). The overall response rate was moderate 56% and the response rate to individual questions was even lower. However, 30% of those who responded did not participate routinely in the care of hip fracture patients and it might be that this proportion was even higher among those who did not respond. On the other hand, the response rate was much greater among specialists than among trainees. Thus the results probably reflect the real current clinical practice, since the practice of a resident might not be as established and stable as that of a specialist.

## Conclusions

According to the present survey preoperative analgesia of hip fracture patients was mainly by systemic paracetamol and oxycodone, and epidural infusion or other regional techniques were used infrequently despite evidence of their beneficial effects. A vast majority of respondents applied spinal block for intraoperative anesthesia. Epidural infusion of a local anesthetic with or without an opioid was commonly employed for postoperative analgesia, although a fair number of respondents used routinely only paracetamol, NSAIDs and opioids. The survey suggests that the impact of more individualised analgesic techniques, both preoperatively and postoperatively, should be investigated in further studies.

## Competing interests

No external funding and no competing interests declared.

## Authors’ contributions

MK, KK, JJ, TT and KL participated and made substantial contributions to the design of the study and drafted the manuscript. SK carried out the most of the work with the Webropol survey and sent the invitations to participate. SK performed the statistical analysis. MK, KK, JJ, TT, SK and KL coordinated the study and were involved in the analysis and interpretation of data. All authors read and revised the manuscript critically and approved the final manuscript.

## Pre-publication history

The pre-publication history for this paper can be accessed here:

http://www.biomedcentral.com/1471-2253/12/14/prepub
